# Phenotypic relationship and repeatability of methane emissions and performance traits in beef cattle using a GreenFeed system

**DOI:** 10.1093/jas/skac349

**Published:** 2022-10-21

**Authors:** Clodagh V Ryan, Thierry Pabiou, Deirdre C Purfield, Stephen Conroy, Stuart F Kirwan, John J Crowley, Craig P Murphy, Ross D Evans

**Affiliations:** Irish Cattle Breeding Federation, Ballincollig, Co. Cork, Ireland; Department of Biological Sciences, Munster Technological University, Bishopstown, Co. Cork, Ireland; Irish Cattle Breeding Federation, Ballincollig, Co. Cork, Ireland; Department of Biological Sciences, Munster Technological University, Bishopstown, Co. Cork, Ireland; Irish Cattle Breeding Federation, Ballincollig, Co. Cork, Ireland; Animal Bioscience Research Centre, Teagasc Grange, Dunsany, Co. Meath, Ireland; AbacusBio Ltd., Dunedin 9016, New Zealand; Department of Agriculture, Food and Nutritional Science, University of Alberta, Edmonton, AB, T6G2R3, Canada; Department of Biological Sciences, Munster Technological University, Bishopstown, Co. Cork, Ireland; Irish Cattle Breeding Federation, Ballincollig, Co. Cork, Ireland

**Keywords:** beef cattle, correlation, GreenFeed, greenhouse gas, methane, repeatability

## Abstract

Rumen methanogenesis results in the loss of 6% to 10% of gross energy intake in cattle and globally is the single most significant source of anthropogenic methane (CH_4_) emissions. The purpose of this study was to analyze greenhouse gas traits recorded in a commercial feedlot unit to gain an understanding into the relationships between greenhouse gas traits and production traits. Methane and carbon dioxide (CO_2_) data recorded via multiple GreenFeed Emission Monitoring (GEM), systems as well as feed intake, live weight, ultrasound scanning data, and slaughter data were available on 1,099 animals destined for beef production, of which 648 were steers, 361 were heifers, and 90 were bulls. Phenotypic relationships between GEM emission measurements with feed intake, weight traits, muscle ultrasound data, and carcass traits were estimated. Utilization of GEM systems, daily patterns of methane output, and repeatability of GEM system measurements across averaging periods were also assessed. Methane concentrations varied with visit number, duration, and time of day of visit to the GEM system. Mean CH_4_ and CO_2_ varied between sex, with mean CH_4_ of 256.1 g/day ± 64.23 for steers, 234.7 g/day ± 59.46 for heifers, and 156.9 g/day ± 55.98 for young bulls. A 10-d average period of GEM system measurements were required for steers and heifers to achieve a minimum repeatability of 0.60; however, higher levels of repeatability were observed in animals that attended the GEM system more frequently. In contrast, CO_2_ emissions reached repeatability estimates >0.6 for steers and heifers in all averaging periods greater than 2-d, suggesting that cattle have a moderately consistent CO_2_ emission pattern across time periods. Animals with heavier bodyweights were observed to have higher levels of CH_4_ (correlation = 0.30) and CO_2_ production (correlation = 0.61), and when assessing direct methane, higher levels of dry matter intake were associated with higher methane output (correlation = 0.31). Results suggest that reducing CH_4_ can have a negative impact on growth and body composition of cattle. Methane ratio traits, such as methane yield and intensity were also evaluated, and while easy to understand and compare across populations, ratio traits are undesirable in animal breeding, due to the unpredictable level of response. Methane adjusted for dry matter intake and liveweight (Residual CH_4_) should be considered as an alternative emission trait when selecting for reduced emissions within breeding goals.

## Teaser

This research aims to emphasize the need for a consistent protocol with regard to methane measurement and data handling and highlight the relationships between methane and production traits.

## Introduction

Rumen methanogenesis results in the loss of 6% to 10% of gross energy intake in cattle and globally is the single most significant source of anthropogenic methane (CH_4_) emissions (Pinares-Patiño et al., 2013). Reducing CH_4_ emissions, therefore, not only improves the environmental sustainability but also the efficiency of both dairy and beef cattle production systems. As such, numerous mitigation strategies for the reduction of CH_4_ have been developed, including the use of feed additives (Gao et al., 2011; Smith et al., 2020; Almeida et al., 2021), nutritional management practices such as increased feed quality (Beauchemin et al., 2008), indirect genetic selection, for example, residual feed intake to reduce methane emissions per kilogram of product and animal performance improvements in milk yields and meat production (O’Brien et al., 2016; Thompson and Rowntree, 2020), and direct genetic selection for reduced emissions (Donoghue et al., 2016a; González-Recio et al., 2020; Lassen and Difford, 2020; de Haas et al., 2021; Manzanilla-Pech et al., 2021). However, before mitigation strategies relating to genetic selection can be implemented, a clear understanding of the CH_4_ phenotype and associated measurement methods is first required.

Genetic selection for reduced emissions proves to be a favorable CH_4_ mitigation strategy due to changes being permanent, cost-effective, and cumulative (de Haas et al., 2021). Nevertheless, despite the importance of CH_4_, it remains excluded from breeding goals primarily due to the expense of requiring large amounts of emission data for its implementation. Respiration chambers, which are viewed as the gold standard CH_4_ measurement approach, are prohibitive for obtaining measurements on the large number of cattle needed for the generation of genetic evaluations due to their substantial capital outlay, high labor costs, and negative impact on animal behavior (Garnsworthy et al., 2019). In 2015, Hammond et al. developed the GreenFeed emission monitoring (GEM) system as an alternative low capital outlay, precise, and robust technique to measure enteric CH_4_ emissions with higher cattle throughput for greenhouse gas mitigation purposes. The GEM system works as a noninvasive system that estimates daily CH_4_ and carbon dioxide (CO_2_) production by measuring gas concentrations and airflow from an animal’s breath upon their visit to a GEM unit. While respiration chambers continue to remain the benchmark for CH_4_ measurement of livestock, strong correlations (0.81 (S.E. 0.10)) between respiration chambers and GEM systems (Garnsworthy et al., 2019) suggest that GEM are a suitable low-cost alternative to measure CH_4_.

Although there is some debate surrounding the reliability of GEM systems (Doreau et al., 2018), the aggregation of multiple measurements over 7–14 days has been shown to result in low variability and high repeatability of emission concentrations (Manafiazar et al., 2017). Manafiazar et al. (2017) also showed that GEM systems consistently ranked animals in terms of CH_4_ emissions. However, as CH_4_ concentrations vary with visit number and duration at the GEM system, there is a large disparity between GEM system protocols in existing CH_4_ emission measurement studies, resulting in implications for data comparison across studies (Della Rosa et al., 2021). Disparity in GEM system protocol also has consequences for analysis within studies, especially for genetic parameter estimation that is impacted by within-method repeatability. As CH_4_ emissions vary throughout the day due to diurnal variation, and from day to day due to changing biological state, the number of measurements and timing of sampling required to obtain a representative daily CH_4_ emissions value is relatively unknown. Therefore, establishment of a protocol for utilization of GEM systems with respect to visit number and duration is paramount to future GEM system data analysis.

Factors such as feeding activity, feed intake, and physiological state, such as growth rate, also impact CH_4_ emissions (Cottle et al., 2015). The relationship between CH_4_, feed intake, and growth indicators such as bodyweight, carcass weight (CW), and ultrasound muscle measurements has been well described using respiration chamber emission measurements (Herd et al., 2014), but limited studies have been completed with large volumes of GEM system measurements across a substantial number of animals.

Therefore, the objective of this study was to use a large quantity of GEM system data from a performance test facility to: 1) assess the impact of animal utilization of GEM systems on CH_4_ and CO_2_ patterns; 2) analyze the phenotypic relationships between GEM emission measurements with feed intake, weight traits, muscle ultrasound data, and carcass traits; and 3) assess the repeatability of GEM emission measurements when averaged across varying time periods. The results from this study will help to establish a standard operating procedure for data handling with respect to generating genetic evaluations for CH_4_ emissions using GEM measurements.

## Materials and Methods

The data used in the present study were obtained from a preexisting database managed by the Irish Cattle Breeding Federation (ICBF). Therefore, it was not necessary to obtain animal care and use committee approval in advance of conducting this study.

### Data

CH_4_ and CO_2_ flux measurements were recorded on 1,099 animals destined for slaughter between 2018 and 2021 as part of progeny performance testing in the Gene Ireland Progeny Performance Test Centre (https://www.icbf.com/?page_id=12900) located in Tully, Co. Kildare, Ireland. All animals were purchased in groups, referred to as intakes; in total, there were 27 intakes of unisex animals with an average intake size of 38.6 animals (SD = 17.22, min. = 10, max. = 72). Animals ranged from 368 days to 910 days of age at the time of entry to the test center and consisted of steers (*n* = 648), heifers (*n* = 361), and young bulls (*n* = 90). All cattle were grouped in pens of 25 animals according to their sex, liveweight, and breed. Breed breakdown of the animals included in this study, by sire and dam breed, are described in Supplementary Table S1. Additional measurements such as feed intake, liveweight, carcass data, and ultrasound muscle measurements were also recorded on all animals as detailed below.

### Methane, CO_2_, and feed intake measurement

Upon arrival to the test center, all 1,099 animals underwent acclimatization periods to adjust to the feeding system, GEM system, and environment. Each pen was equipped with 10 automatic feed stations (RIC Feed-Weigh Trough, Hokofarm Group BV, Marknesse, The Netherlands) to measure feed intake. Each station provided ad libitum access to feed. Acclimatization for feed intake was deemed complete when animals had spent between 21 and 30 days using the feed intake boxes. Each pen also had ad libitum access to clean, fresh water via five water troughs spaced evenly between the automatic feed stations. Supplementary Figure S1 provides further details of pen layout. Full details of acclimatization management for feed intake are detailed by Kelly et al. (2019).

In addition to the feed stations, each pen was also equipped with one GEM system to record CH_4_ and CO_2_ emissions of the animals. Gas measurement acclimatization was monitored by the number of visits per day to the GEM system and animals were considered acclimatized when each animal in the pen had used the GEM system for three consecutive days. To prevent other animal interference in gas measures, each GEM had a side gate to ensure only one animal was present at each measurement. The side gate was absent in the acclimatization period to all the animals become familiar with the GEM system. Both the automatic feed stations and GEM systems monitored animal visitation using a radio frequency identification (RFID) tag of each individual (EID tag, Allflex Livestock Intelligence, Dallas, TX). GEM systems calculate the concentration of gas emitted using background gas concentration, calibration coefficients, and the differential concentration of gas during the animal’s visit to the GEM system. Full details of calibration and GEM system measurement are described elsewhere (Hammond et al., 2015). Upon entering the GEM system, the system dispensed 30 g of feed every 30 s, thus enticing the animal to stay at the machine. Each animal was permitted a maximum of six aliquots of feed per 4-h period, allowing the animal to receive a maximum of 1,080 g of feed from the GEM system in a 24-h period. Visits were considered complete when the animal had exited the GEM system and the RFID tag was no longer in range.

### Diet

A total mixed ration (TMR) of approximately 13.95% hay, 45.35% concentrates, and 40.7% water was provided to the steers and heifers once per day during the test period with a paddle mixer wagon. Insentec feed station refill times for Monday to Friday were between 09:00 and 11:00, whereas on Saturday and Sunday, the feed stations were refilled from approximately 08:30 to 13:00. The TMR was estimated to have a dry matter of 51% and a metabolizable energy value of 12.1 MJ/kg DM. Young bulls were fed concentrates ad libitum, based on consumption of feed during the acclimatization period. All young bulls started the acclimatization period on 5 kg fresh weight of concentrates and increased by 0.5 kg fresh weight per day until ad libitum levels were reached. In addition, to maintain rumen health, a daily fixed rate of 2 kg fresh weight of hay was provided to all young bulls. GEM system feed storage bins were filled with concentrates every second day. The concentrates offered within the GEM system to all test animals had dry matter of 86% and a metabolizable energy concentration of 14.1 MJ/kg DM.

### Liveweight, ultrasound measurement, and carcass data

Liveweight measurements were available for all animals in the study. Prior to acclimatzsation, all animals were weighed on arrival at the center. Animals also had a final liveweight recorded three days prior to slaughter. The final liveweight, recorded prior to slaughter was used in the present study. Ultrasound measurements were also recorded on all bulls, 610 steers, and 351 heifers during the test period to measure ultrasound fat depth (FD), eye muscle depth (EMA), and intramuscular fat (IMF), using an Esaote-Pie Medical Aquila PRO Vet ultrasound scanner with a 3.5 MHz transducer head. Full description of the ultrasound measurements were previously reported by Kelly et al. (2019). Carcass data was recorded at slaughter in the abattoir. CW was recorded as the cold dressed carcass weight recorded, on average, 2-h post-slaughter. Carcass conformation (CC) and carcass fat (CF) class were obtained from the abattoir using video image analysis from a mechanical grading system (Pabiou et al., 2011). CC was defined by the EUROP system and represented by the letters E, U, R, O, and P, where E represents the best conformation and P represents the worst conformation (Englishby et al., 2016). Each conformation class was subdivided into three categories (+, =, −), resulting in a 15-point scale for CC. CF classes were presented on a scale from 1 to 15, where 1 represents the least level of fat cover and 15 represents the greatest level of fat cover on the carcass.

### Data edits

The test period in this study varied by intake group and consisted of a start date, when all animals in the pen were trained to use the GEM system, and an end date, immediately prior to slaughter. Test period length was driven by management decisions taken in the commercial feedlot and varied by intake group ranging from 20 to 83 days. From the initial dataset of 1,099 animals, 18 individual animals failed to use the GEM system while on test; all data from these animals were removed from the analysis. From the available 246,727 individual animal visits from the GEM system, emission data from visits outside of the defined test period (*n* = 1,24,838) were excluded from analysis due to multiple animals having access to the GEM machine simultaneously. GEM system visit accumulation data were collated based on sex and the number of visits accumulated per day across the test period was calculated.

Emission measurements from the GEM systems underwent an initial quality control by the technology provider C-Lock prior to reporting to ICBF, which involved the removal of all animal visits under 2 min duration. In addition, CH_4_ and CO_2_ measurements less than zero or in the top or bottom 1% of the data were also removed (*n* = 4,214) from the reported data. To ensure all feed intake data directly related to the same period as GEM system measurements, feed intake records pertinent to GEM system visits were retained (*n* = 123,366). After all edits, GEM emission measurements, feed intake, liveweight data, and carcass data were available on 1081 animals of which 510 were steers, 361 were heifers, and 90 were young bulls.

### Trait definitions

Methane and CO_2_ were defined as the gas flux measurement recorded by the GEM system in grams per day (Table 1). Each individual GEM CH_4_ and CO_2_ emission measurement was reported in grams per day per measurement, resulting in some animals having multiple CH_4_/CO_2_ grams per day values available. Feed intake measured in kilograms of dry matter intake per day (DMI) was estimated as the combined feed consumed per animal from both GEM systems and automatic feed stations in the 24-h period prior to CH_4_ measurement, thus resulting in an individual dry matter intake per day per animal. Liveweight was recorded twice during the residence of the animals in the feedlot, first, prior to acclimatization, and again, one day prior to slaughter. Ultrasound IMF was recorded as a percentage, estimated from images taken at a lateral position to the animal’s spine at the 13th thoracic rib. Eye muscle area was recorded as a single millimeter measurement at the deepest point of the eye muscle at the third lumbar vertebra on top of the loin. FD was the millimeter measurement averaged from FDs recorded at the third lumbar vertebrae and at the 13th thoracic rib. Four additional CH_4_ traits were defined and analyzed in this study. These included methane yield (MY), methane intensity relative to liveweight (MIL), methane intensity relative to carcass weight (MIC), and residual CH_4_ (RCH_4_). MY, a ratio trait, was derived as g CH_4_/kg DMI, calculated from the final 10-d CH_4_ average and the corresponding 10-d DMI average based on the 24-h period prior to CH_4_ measurement from the same period. MIL and MIC were ratio traits, defined as the final 10-d GEM system CH_4_ measure expressed as grams of CH_4_ per kilogram of liveweight or CW, respectively. The liveweight in MIL utilized the final liveweight recorded prior to slaughter, reflective of the final 10 d of test. Residual CH_4_ (RCH_4_) was estimated by regression using Statistical Analysis System 9.4 software (SAS 9.4, SAS Institute, Cary, NC, USA). The linear model procedure, Proc GLM, was used to correct CH_4_ for DMI and LW, that is, the final 10-d average CH_4_, and 10-d average DMI based on DMI consumed within the 24-h period prior to CH_4_ measurement, and LW prior to slaughter.

**Table 1.** Trait definitions

Trait name	Abbreviation	Unit	Definition
Methane	CH_4_	g/day	Methane in grams emitted per day, based on an individual GEM system measurement. Animals can have multiple records per day.
Carbon dioxide	CO_2_	g/day	Carbon dioxide in grams emitted per day, based on an individual GEM system measurement. Animals can have multiple records per day.
Dry matter intake	DMI	kg/day	Dry matter intake in kilograms per individual day during methane measurement.
Live weight	LW	kg/day	Kilograms of liveweight on a specific day during the test period.
Eye muscle area	EMA	mm	Millimeter measurement at single deepest point of the eye muscle at the third lumbar vertebra on top of the loin.
Fat depth	FD	mm	Millimeter measurement average of all fat depths recorded at the third lumbar vertebrae and at the 13th thoracic rib.
Intramuscular fat	IMF	%	Percentage estimated from images taken at a lateral position to the animal’s spine at the 13th thoracic rib.
Carcass weight	CW	kg	Kilograms of dressed carcass, weighed 2 h post slaughter.
Carcass conformation	CC	15-point scale	EUROP classes subdivided into three categories, resulting in a 15-point scale.
Carcass fat	CF	15-point scale	Scale from 1 to 15, where 1 represents the least level of fat cover and 15 represents the greatest level of fat cover.
Methane yield	MY	g/kg	Ratio trait of methane in grams divided by kilograms of dry matter intake per day.
Methane intensity (liveweight)	MIL	g/kg	Ratio trait of methane in grams divided by kilograms of liveweight.
Methane intensity (carcass weight)	MIC	g/kg	Ratio trait of methane in grams divided by kilograms of carcass weight.
Residual methane	RCH_4_	g/day	Methane adjusted for dry matter intake and live weight.

### Statistical analysis

Data in this study were analyzed using the Statistical Analysis System 9.4 package (SAS 9.4, SAS Institute, Cary, NC, USA). Due to the repeated nature within animal and within and across days on test, data recorded by the GEM system (multiple daily grams per day values), CH_4_ and CO_2_ data were averaged by animal across six varying time periods (1-d, 2-d, 4-d, 5-d, 10-d, and 15-d). For each time period, the average DMI was also estimated. Additionally, for CH_4_ and CO_2_, within-day repeatability was estimated between each animal visit.

To ensure a fair comparison between test period lengths, phenotypic repeatability for CH_4_, CO_2_, and DMI was estimated for each time period (within-day, 1-d, 2-d, 4-d, 5-d, 10-d, and 15-d) using only animals who were on test for a minimum of 30 d (*n* = 752). No restriction was applied to maximum test length to maximize the number of animals included in the analysis. However, data analyzed in repeatability analysis was restricted to records observed in the first 30 d of the test period to ensure completion of two 15-d time periods. The phenotypic repeatability analysis required the estimation of the variances and covariances across time periods using PROC MIXED (SAS 9.4, SAS Institute, Cary, NC, USA), and the model applied was:


$$\begin{array}{l} y=Xb+Za+e \end{array}$$

where **y** is a vector for trait of interest (CH_4_, CO_2_, or DMI), **b** is a vector of fixed effects including contemporary group, time period (within-day, 1-d, 2-d, 4-d, 5-d, 10-d, and 15-d), time-of-day of measurement (only included in the model for within-day analysis for CH_4_ and CO_2_); **a** is a random repeated effect of animal; and **e** is the random residual term. **X** and **Z** are the corresponding incidence matrices connecting phenotypes to effects.

Similar to Renand and Maupetit (2016), the structure of the covariance matrix across time periods assumed constant among-animal (σ^2^_i_) and within-animal (σ^2^_r_) variances. The covariance between measures at any two time periods was σ^2^_i_, and the variance of measures at each time period was (σ^2^_i_ + σ^2^_r_). Repeatability coefficients determined from the PROC MIXED output were calculated as repeatability = σ^2^_i_/(σ^2^_i_ + σ^2^_r_).

Repeatability analysis was constructed by sex initially, and additionally stratified into three categories based on total number of visits to the GEM system during the test period; the top 33% of GEM system visitors within each sex were categorized as “Good Visitors,” the middle 33% of each sex were categorized as “Average Visitors” and the bottom 33% of visitors for each sex were categorized as “Poor Visitors.”

Phenotypic correlations between all traits (CH_4_, CO_2_, DMI, MY, MIL, MIC, RCH_4_, LW, EMA, FD, IMF, CW, CC, and CF) were calculated using SAS Proc Corr using single average CH_4_, CO_2_, and DMI measurements based on the last 10-d average value for animals who had GEM system measurements within 10 d of slaughter (*n* = 762). All correlations were adjusted for age and contemporary group, as previously defined, through the inclusion of an age and contemporary group interaction.

## Results

Table 2 displays descriptive statistics for each trait measured. Mean test length varied from 40.6 d in heifers, 51 d in steers and 66.9 d in bulls due to group specific management decisions and ranged from 16 to 84 d. On average 247 g/day of CH_4_ and 9376 g/day of CO_2_ were emitted per animal. The raw mean CH_4_ was lower in the bull cohort (156.9 g/day); however, due to diet differences the mean CH_4_ emissions for bulls were not directly comparable to steer and heifer means. Considerable variation was detected in CH_4_ emissions within each sex; the coefficient of variation (CV) ranged from 25.08% in steers, 25.33% in heifers, and 35.68% in the bull cohort. A similar trend was observed for CO_2_, with bulls having a higher CV (14.86%) than heifers (11.93%) and steers (11.26%). Dry matter intake differences between bulls vs. heifers and steers were not directly comparable as already mentioned due to diet differences, however, the CV of all three sexes were similar, 20.0%, 19.48%, and 27.88%, respectively. LW varied from 553.8 kg for heifers to 667.2 kg for bulls (Table 2). Muscle depth ranged from 42 mm to 103 mm, FD from 2 mm to 11 mm, and IMF from 2% to 8% across steers, heifers, and bulls. CW for all animals ranged from 202 kg to 506 kg, and CC grades ranged from 3 (P+) to 14 (E=), reflective of the multibreed population captured in the intake runs. Bull CF average was lower than steers and heifers at 5.9 compared with 7.2 and 7.6, respectively (Table 2).

**Table 2.** Raw means with standard deviations in parentheses of GEM system measurements, feed intake and performance traits of steers, heifers, and bulls

	Steers	Heifers	Bulls
Number of animals	630	361	90
Age, day	576.7 (97.53)	455.6 (43.61)	426.2 (27.87)
Test period length, day	51.0 (21.44)	40.6 (23.02)	66.9 (4.95)
GEM Visits across test	132.5 (72.46)	96.0 (89.28)	41.3 (31.47)
Methane, g/day	256.1 (64.23)	234.7 (59.46)	156.9 (55.98)
Carbon dioxide, g/day	9704.9 (1250.69)	8595.4 (1163.52)	9338.2 (1411.48)
Dry matter intake, kg/day	13.1 (2.62)	11.6 (2.26)	11.8 (3.29)
Live weight, kg	662.5 (62.62)	553.8 (58.97)	667.2 (65.10)
Eye muscle area, mm	70.6 (10.05)	73.1 (7.54)	82.2 (7.76)
Fat depth, mm	5.2 (1.58)	5.5 (1.82)	4.0 (1.34)
Intramuscular fat, %	6.6 (1.12)	6.1 (1.38)	6.1 (1.04)
Carcass weight, kg	357.3 (39.98)	305.0 (35.68)	392.7 (41.63)
Carcass conformation	7.8 (2.36)	8.8 (1.66)	10.6 (1.66)
Carcass fat	7.2 (1.43)	7.6 (1.87)	5.9 (1.39)
Methane yield, g CH_4_/kg DMI	19.8 (4.04)	20.9 (4.04)	15.1 (8.67)
Methane intensity (liveweight), g CH_4_/kg LW	0.4 (0.07)	0.4 (0.08)	0.3 (0.10)
Methane intensity (carcass weight), g CH_4_/kg CW	0.7 (0.15)	0.8 (0.16)	0.5 (0.18)
Residual methane, g/day	0.0 (45.09)	0.0 (38.12)	0.0 (49.31)

For the methane ratio traits (MY, MIL, and MIC), it should be noted that lower values are desirable as these traits are estimated to reduce GHG emissions per unit of feed, LW, and CW. Mean MY (g CH_4_/kg DMI) was 19.8 g/kg, 20.9 g/kg, and 15.1 g/kg for steers, heifers, and bulls, respectively and ranged from 10.9 g/kg to 191.8 g/kg (Table 2). Methane intensity traits ranged from 0.1 to 0.7 g CH_4_/kg LW for MIL and from 0.2 to 1.5 g CH_4_/kg CW for MIC. CV for MY varied by sex from 19.4% for heifers to 57.6% for bulls. Smaller ranges of CV were observed for MIL and MIC, from 19.2% for steers to 31.7% for bulls and 21.3% for heifers to 32.3% for bulls for MIL and MIC respectively. By definition, the mean RCH_4_ in g/day for all sexes was 0.0 g/day, but the standard deviation varied between sexes from 38.12 g/day for heifers, 45.09 g/day for steer, and 49.31 g/day for young bulls.

### GEM systems and animal visitation

**Figure 1.**
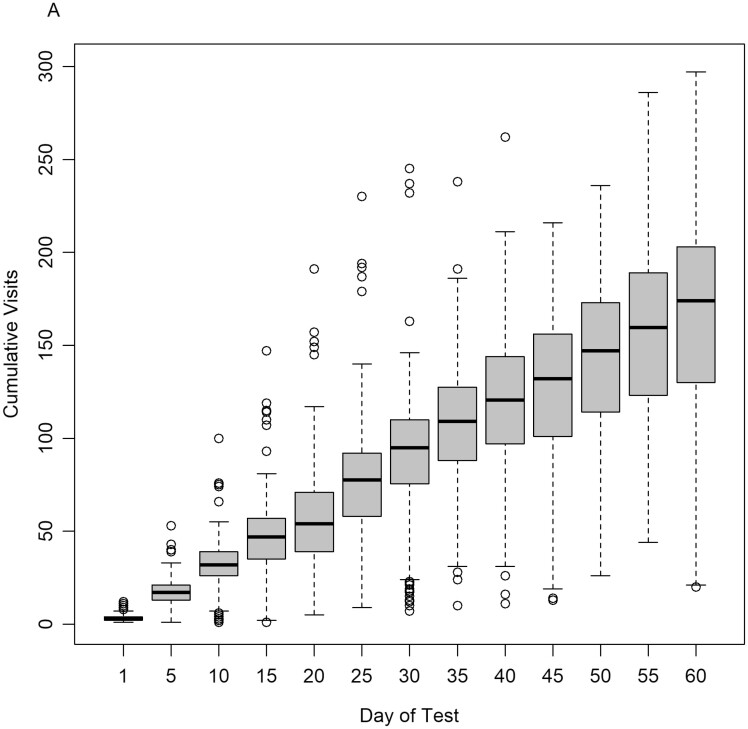
Visit accumulation data of animals (*n* = 412) in the study across the first 60 d on test.

The average number of visits to the GEM system per animal throughout the test period was considerably lower for the young bulls (41.3) in comparison to the steer (132.5) and heifer (96.0) cohorts (Table 2). The average length of a visit to a GEM system was 3.47 min and the mode of all visit hours was 00:00, meaning most visits to the GEM system occurred at midnight, with the next most frequent visit hour being 7:00. The number of GEM system visits per animal included in the repeatability analysis which restricted records observed in the first 30 d of the test period to ensure completion of two 15-d time periods, ranged from 5 to 349 visits during the test period. Figure 1 shows the GEM system visit accumulation of all animals. On average, it took 17.28 days (SD 2.76) for animals to achieve a minimum of 50 visits as recommended by Cottle et al. (2015) and Manafiazar et al. (2017). However, visit frequency to the GEM system per animal varied across the test period with periods of sparse and frequent visitation. One steer achieved 53 visits to the GEM system in the first 5 d on test, while one bull had only one visit in the first 10 d on test but accumulated a further nine visits by the 15th d on test. The largest number of visits achieved by a single animal during a 60-d test period was 297 visits to the GEM system, with the poorest animal on a 60-d test achieving 20 visits.

**Figure 2.**
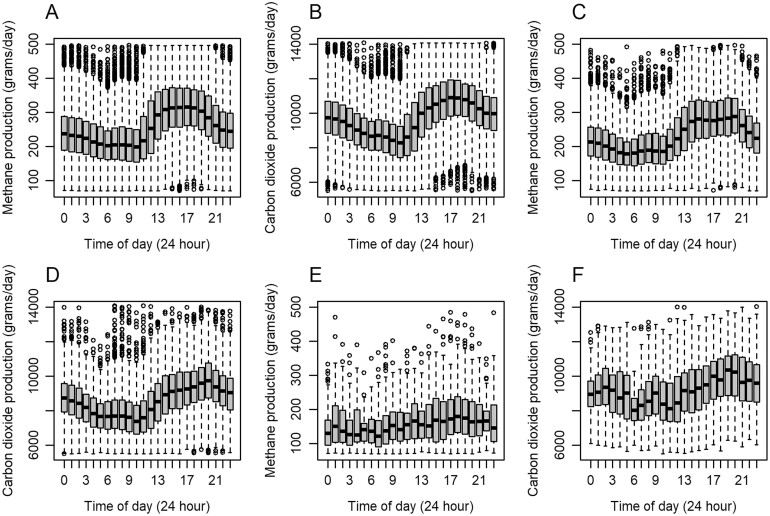
Pattern of A) methane production of steers, B) carbon dioxide production of steers, C) methane production of heifers, D) carbon dioxide production of heifers, E) methane production of bulls, F) carbon dioxide production of bulls observed from GreenFeed emission monitoring system measurements.

Figure 2 displays the effect of time of day on CH_4_ and CO_2_ animal emissions per sex. Lowest levels of CH_4_ were observed between 5:00 and 10:00 with a mean CH_4_ of 204 g/day, whereas the highest levels of CH_4_ emissions were observed between 15:00 and 18:00 with a mean CH_4_ of 302 g/day. Additionally, a similar diurnal pattern was observed for CO_2_ recorded by the GEM system in steers and heifers (Figure 2A–D). Lowest levels of CO_2_ were also observed between 5:00 and 10:00 with a mean CO_2_ of 8,427 g/day whereas highest levels of CO_2_ were observed between 15:00 and 20:00 with a mean CO_2_ of 10,299 g/day. No distinctive diurnal pattern for both CO_2_ and CH_4_ was observed in the bull cohort (Figure 2E and F).

### Phenotypic correlations between GEM system measurements and performance traits

Phenotypic correlations, which were adjusted for animal age and contemporary group, were positive between CH_4_ and all measured traits (Table 3). A strong phenotypic correlation of 0.61 existed between CH_4_ and CO_2_, suggesting that higher emissions of CH_4_ were associated with higher emissions of CO_2_. Carbon dioxide was similarly positively correlated with all measured performance traits with a strong positive correlation detected between CO_2_ and LW (0.61) and a moderate correlation between CO_2_ and CW (0.52). Dry matter intake had a moderate correlation with both CH_4_ (0.31) and CO_2_ (0.57).

**Table 3.** Phenotypic correlations between GreenFeed emission monitoring system measurements, dry matter intake, ultrasonic muscle measurements, liveweight traits, and carcass traits, adjusted for age

	CH_4_	CO_2_	DMI	MY	MIL	MIC	RCH_4_	LW	EMA	FD	IMF	CW	CC
CO_2_	0.61^a^												
DMI	0.31^a^	0.57^a^											
MY	0.56^a^	NS	−0.49^a^										
MIL	0.86^a^	0.31^a^	NS	0.65^a^									
MIC	0.84^a^	0.32^a^	0.07^a^	0.61^a^	0.97^a^								
RCH_4_	0.63^a^	0.27^a^	NS	0.50^a^	0.62^a^	0.60^a^							
LW	0.30^a^	0.61^a^	0.58^a^	−0.15^a^	−0.21^a^	−0.18^a^	NS						
EMA	NS	0.14^a^	0.08^a^	NS	−0.10^a^	−0.19^a^	NS	0.30^a^					
FD	0.10^a^	0.20^a^	0.30^a^	–0.08^a^	–0.07^a^	NS	NS	0.34^a^	NS				
IMF	0.14^a^	0.19^a^	0.24^a^	NS	NS	0.08^a^	NS	0.25^a^	−0.10^a^	0.36^a^			
CW	0.24^a^	0.52^a^	0.46^a^	−0.12^a^	−0.22^a^	−0.28^a^	NS	0.91^a^	0.46^a^	0.22^a^	0.13^a^		
CC	NS	0.12^a^	NS	NS	−0.13^a^	−0.25^a^	NS	0.28^a^	0.52^a^	NS	−0.12^a^	0.53^a^	
CF	0.17^a^	0.28^a^	0.35^a^	−0.08^a^	NS	NS	NS	0.44^a^	NS	0.67^a^	0.38^a^	0.30^a^	NS

^a^ *P* value <0.05.

^1^ CH_4_, methane; CO_2_, carbon dioxide; DMI, dry matter intake; MY, methane yield; MIL, methane intensity (liveweight); MIC, methane intensity (carcass); RCH_4_, residual methane; LW, liveweight; EMA, eye muscle area; FD, fat depth; IMF, intramuscular fat; CW, carcass weight; CC, carcass conformation; CF, carcass fat.

^2^ NS, not significantly different from zero (*P* value ≥0.05).

MY had a moderate correlation with CH_4_ (0.56), a non-significant correlation with CO_2_, and a moderate negative correlation with DMI (−0.49). MY was also weakly negatively correlated with LW, FD, CW, and CF. As expected, due to trait definition, methane intensity traits (MIL, MIC) had a correlation of near unity (0.97) and were both strongly positively correlated with CH_4_, 0.86 and 0.84, respectively (Table 3). Strong correlations were observed between RCH_4_ and CH_4_ (0.63), MY (0.50), MIL (0.62), and MIC (0.60). The strong positive correlations between RCH_4_ and CH_4_, MY, MIL, and MIC are reflective of the moderate relationship between CH_4,_ and the other trait used in the derivation of MY, MIL, and MIC. RCH_4_, which was corrected for both DMI and LW, was weakly negatively correlated (−0.01) with DMI; however this was not statistically significant (*P* > 0.05). Phenotypic correlations between RCH_4_ and LW, EMA, FD, IMF, CW, CC, and CF were not different (*P* > 0.05) from zero.

### Phenotypic repeatability of GEM system measurements and DMI

**Figure 3.**
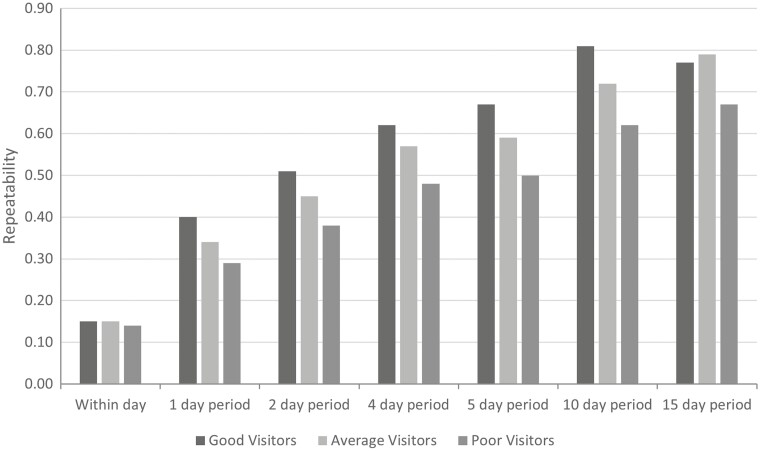
Phenotypic repeatability of methane in steers, based on visit frequency. The top 33% of GreenFeed emission monitoring system visitors (steers) were categorized as good visitors, the middle 33% were categorized as average visitors, and the bottom 33% of visitors were categorized as poor visitors.

To ensure the majority of animals were represented in each repeat period (within-day, 1-d, 2-d, 4-d, 5-d, 10-d, and 15-d), phenotypic repeatability analysis included only animals who were on test for a minimum of 30 d (*n* = 752). Repeatability of all GEM emission measurements and DMI increased as the length of time of the averaging period increased (Table 4). Repeatability of CH_4_ from GEM system measurements ranged from 0.14 to 0.74 depending on the animal sex and length of averaging period (Table 4). Repeatability of CO_2_ was slightly higher than CH_4_, ranging from 0.21 to 0.82. While repeatability of GEM system measurements were low initially, repeatability measures >0.6 for CH_4_ were achieved with a 10-d averaging repeat period in steers and heifers. In contrast, CO_2_ reached repeatability levels >0.6 for steers and heifers in all averaging periods greater than 2-d (Table 4). Due to the poor GEM visit frequency in the bull cohort, repeatability of GEM system measurements in bulls were much lower than the steer and heifer cohorts. Animal visit frequency impacted the phenotypic repeatability, with the repeatability of good visitor animals being higher than that of the poor visitor animals (Figure 3).

**Table 4.** Phenotypic repeatability of methane, carbon dioxide, and dry matter intake through different averaging periods for steers, heifers, and bulls

	Steers					Heifers					Bulls				
Number of animals	467					195					90				
Repeat period	*n* ^1^	CH_4_	CO_2_	*n* ^1^	DMI	*n* ^1^	CH_4_	CO_2_	*n* ^1^	DMI	*n* ^1^	CH_4_	CO_2_	*n* ^1^	DMI
Within day	39,430	0.16	0.21	N/A	N/A	13,391	0.14	0.24	N/A	N/A	2,039	0.20	0.22	N/A	N/A
1 d period	11,599	0.34	0.44	12,209	0.39	3,796	0.32	0.50	4,236	0.43	1,008	0.24	0.34	2,310	0.45
2-d period	6,055	0.44	0.55	6,105	0.56	2,032	0.38	0.58	2,118	0.59	647	0.29	0.37	1,155	0.60
4-d period	3,307	0.55	0.65	3,256	0.65	1,126	0.45	0.69	1132	0.70	425	0.34	0.47	616	0.67
5-d period	2,557	0.58	0.67	2,442	0.72	9,03	0.48	0.71	876	0.72	374	0.26	0.47	462	0.71
10-d period	1,328	0.70	0.76	1,221	0.81	462	0.61	0.78	438	0.84	242	0.32	0.52	231	0.79
15-d period	928	0.74	0.80	814	0.85	310	0.69	0.82	292	0.84	176	0.32	0.61	154	0.82

^1^ Number of observations in repeatability analysis.

^2^ N/A, not available.

As CH_4_ and CO_2_ records were collected separately to DMI, a different number of records contributed to the repeatability analysis for DMI, with the maximum number of records included in the DMI 1-day repeatability analysis (*n* = 39,430). Dry matter intake repeatability across the repeatability periods was higher than those achieved with GEM system measurements, ranging from 0.39 to 0.84 across steers, heifers, and bulls. Due to the high number of animal visits to the feed stations, strong levels of repeatability (>0.60) were achieved in the 4-d repeat period for both steers and heifers (Table 4). In comparison, the bull cohort achieved acceptable levels of repeatability for DMI in the 2-d averaging period, which may reflect the smaller sample size, or diet differences.

## Discussion

There has been an increasing demand to measure CH_4_ and related traits for animals as part of mitigation strategies and to measure these animals in their production environment (Velazco et al., 2016). While respiration chamber studies are the gold standard when it comes to CH_4_ measurements (Donoghue et al., 2016a, 2016b; Arthur et al., 2018), GEM systems facilitate a more cost-effective alternative while permitting animals to accumulate CH_4_ measures in their usual production environment. Numerous previous studies have compared respiration chambers with multiple methods of methane measurement, including GEM systems, sniffer methods, and laser detector methods (Hammond et al., 2015, 2016; Arbre et al., 2016; Difford et al., 2018; Doreau et al., 2018; Garnsworthy et al., 2019; Zhao et al., 2020; Della Rosa et al., 2021). Strong correlations (0.81) between respiration chambers and GEM systems have been previously established (Garnsworthy et al., 2019) and Doreau et al. (2018) also found differences between respiration chambers and GEM systems to be minor suggesting GEM systems provide reliable emission measurements for groups of animals.

In the present study, the mean CH_4_ production of heifers 234.7 g/d was considerably higher than those previously reported in beef heifers by both Renand et al. (2019) and Alemu et al. (2017) using GEM systems. This increased methane may be reflective of diet or age of the test animals but also may be reflective of the multiple breeds captured within the present study. Rooke et al. (2014) and Flay et al. (2019) reported that methane emissions vary widely by breed despite similar management. While Flay et al. (2019) concluded that Jersey heifers (242 g CH_4_) had lower CH_4_ per day than Holstein Friesian heifers (267 g CH_4_), Flay et al. (2019) also concluded that breed did not affect MY or MIL, which was consistent with results observed by Grainger and Goddard (2004) and Rooke et al. (2014), as trait definitions capture breed differences in DMI. Similar MY means were observed between the steers and heifers in this study (19.8 g/kg DMI and 20.9 g/kg DMI, respectively) and the Holstein Friesians and Jerseys (21.6 g/DMI and 21.9 g/DMI, respectively) in Flay et al. (2019). Slightly higher MIL was observed in Flay et al. (2019) for both Holstein Friesians and Jerseys (0.56 g/kg LW, 0.59 g/kg LW) compared with the multi-breed animals in this analysis (0.2–0.4 g/kg LW); however, the point at which LW was determined differed in Flay et al. (2019), as they utilized a mid-test weight opposed to an end of test weight which used in the present study to derive MIL. The mid-test weight observed in Flay et al. (2019) ranged from 408 kg for Jerseys and 480 kg for Holstein Friesians, compared with 553.8 kg for heifers, 662.5 kg for steers, and 667.2 kg for bulls observed in this study.

### GEM systems and animal visitation

The present study highlighted the vast variation that exists in both animal visit frequency and visit duration to a GEM system. Currently, only 30% of existing GEM studies have a detailed visit duration (Della Rosa et al., 2021). Of the 30 GEM system studies reviewed by Della Rosa et al. (2021), the mean visit duration was 3.4 min, similar to the 3.47 min mean visit duration observed in this study. Della Rosa et al. (2021) also reported a wide range in the number of visits an animal makes to a GEM system across trials, ranging from 6 to 141 visits with a mean of 63 visits. In contrast, the mean number of visits in the present study was much greater for steers (132.5) and heifers (96.0). The greater number of animal visits in this study may be a reflection of the extended test period length, ranging from 20 to 83 days, whereas only 57% of studies reviewed by Della Rosa et al. (2021) had a 15–91 test day length, with a further 30% of studies having sampling periods ranging from 2 to 14 days.

Additionally, unlike Huhtanen et al. (2019), where some animals were housed in a tie-stall system with GEM systems brought to the animal at fixed time points throughout the day, animals in this study were required to visit the GEM system independently, with no human intervention. As visitation to the GEM system is entirely voluntarily, some animals in this study (*n* = 18) continually showed a lack of interest in the GEM systems, and instead relied on the automatic feed stations for all concentrate feed intake. This was particularly true for the bull cohorts that had ad libitum access to concentrates in the Insentec boxes that was of the same specification as that offered in the GEM system and, therefore, demonstrated less desire to visit the GEM system. While it would be desirable to ensure all animals utilized the GEM system fully, the animals in this study were in a commercial feedlot setting, hence, CH_4_ data recording was not the sole aim of producers.

The diurnal pattern of methane can be described as lowest CH_4_ emissions occurring near sunrise and largest emissions occurring near sunset (Van Haarlem et al., 2008). Basarab et al. (2013) suggested a minimum 35 d of feed intake records to achieve moderate within-animal repeatability and capture of distinctive diurnal patterns in relation to DMI; however, for CH_4_ measures in the present study, a 10-d average period achieved moderately strong within-animal repeatability (>0.6) and captured the diurnal pattern. The most frequent time animals in this study visited the GEM system (00:00, followed by 07:00) were largely in agreement with analysis carried out by Alemu et al. (2017), where animals most often visited the machine at midnight and between 6 a.m. and 7 a.m. The quantity of enteric CH_4_ emitted by animals varied throughout the day, highlighting the need for inclusion of time-of-day covariates in future analysis and due to the differences in number of GEM system visits across animals, further consideration needs to be given to data handling for all future analysis and with respect to genetic evaluations.

While numerous studies have been carried out where CH_4_ measurements were collected using GEM systems (Hammond et al., 2015; Arbre et al., 2016; Renand & Maupetit, 2016; Waghorn et al., 2016; Alemu et al., 2017; Manafiazar et al., 2017; Huhtanen et al., 2019; Suybeng et al., 2021), to the authors’ knowledge, this is the first study where data has been collected via a large number of separate GEM systems in a commercial feedlot unit. In total, 10 GEM systems were used in this study, while the majority of existing studies used a single GEM system for all animal records (Arbre et al., 2016; Manafiazar et al., 2017; Doreau et al., 2018), the number of GEM systems has varied from two (Velazco et al., 2016) to four (Suybeng et al., 2021). While the procedure followed by Suybeng et al. (2021) represents an ideal method of having animals measured on multiple GEM systems on multiple occasions to account for machine variability, this was not possible in the commercial feedlot in this study. The resulting variability from having 10 GEM systems in this study was factored in by including the GEM system machine number in contemporary group definitions. Including the GEM system number in contemporary group forms part of the proposed protocol for GEM system data handling.

### Impact of repeatability of GEM system measurements

To date, there is a paucity on the repeatability of emission in the literature, and previous studies have been limited on population size. Determination of the repeatability of a trait allows us to determine if the animals exhibit a consistent pattern over time and also sets the upper threshold for heritability measures. Repeatability measures have been found to differ per measurement, ranging from 0.47 for milk (Johnson and Corley, 1961), 0.72–0.82 for body weight (Pszczola et al., 2018), 0.34 for feed intake (Kelly et al., 2010), and 0.26–0.50 for fecal egg count (Pal and Chakravarty, 2020). In the present study, emission measures were averaged across time periods, thus removing a large proportion of the within-day variance associated with the traits and thus increasing repeatability measures. As such, a repeatability measure of 0.6, which is reflective of the upper heritability estimates of CH_4_ and CO_2_ previously reported elsewhere (Donoghue et al., 2020; van Breukelen et al., 2022) was chosen as a threshold to capture moderately consistent emission pattern.

While repeatability estimates for CH_4_ were similar to previously studies (Arbre et al., 2016; Renand & Maupetit, 2016; Manafiazar et al., 2017), the larger number of animals and different sexes analyzed in this study provides an insight into how repeatability can be impacted by sex and visit frequency. Broadly similar results were achieved in terms of repeatability to that of Renand and Maupetit (2016) for heifers; CH_4_ repeatability results for heifers in this study ranged from 0.32 to 0.69 compared with 0.26–0.67 for similar averaging periods (Renand & Maupetit, 2016). However, in comparison, the steers in the present study had higher repeatability estimates across all time periods (0.34–0.74), suggesting a shorter test period would be sufficient to capture CH_4_ emission patterns for steers than heifers, which is most likely a reflection of their increased behavior to frequent GEM systems.

Additionally, in this study, repeatability of GEM system measurements within day were analyzed, which has not been previously reported. Within-day CH_4_ repeatability, albeit weak (0.14–0.19), suggests need for exploration of repeatability models of individual CH_4_ measures in future genetic analysis. As previously reported by Manafiazar et al. (2017), CO_2_ repeatability estimates were higher than CH_4_ repeatability estimates, ranging from 0.20 within day to 0.82 for a 15-d averaging period. Repeatability estimates for CH_4_ > 0.6 were reached at a 10-d averaging period in this study, suggesting that a minimum of 10 d of GEM system measurements would be sufficient to capture emission patterns. However, this may be dependent on the number of visits per animal within the 10-d period. When assessing repeatability based on animal visit frequency, ‘good visitor’ steers had a repeatability of 0.81 for 10-d average period, compared with ‘poor visitor’ steers, which demonstrated lower repeatability levels of 0.62 for the same averaging period (Figure 3). The disparity of repeatability based on visit frequency highlights the need for cognizance of visit frequency in analysis. In contrast, Renand and Maupetit (2016) suggested 35 d of GEM system recording, in conjunction with feed intake testing, is necessary as extending test duration reduced within-animal variability. However, Renand and Maupetit (2016) acknowledged that if a sufficient acclimatization period was used, by, for example, discarding the initial two weeks, good repeatability of CH_4_ measures would be observed after a further 2 wks, as was seen in the present study. The CV for steers reduced from 34.18% to 19.17% from within-day CH_4_ measures to the 10-d average period, heifer CV reduced from 36.15% to 21.54% and young bull CV reduces from 42.10% to 29.06%. When assessing the 30-d average periods in this study, CV was largely similar to the 10-d average period with a CV of 17.66% for steers, 21.39% for heifers, and 27.0% for young bulls. This suggests that a 10-d average period rather than 30-d average CH_4_ may be sufficient when designing protocols for GEM measurement to ensure adequate repeatability and reduce within-animal variation. However, caution is required when shortening the length of test period as a balance must be struck between the cost associated with a longer test period and the loss of accuracy associated with shorter test periods. It is also important to note that increased levels of repeatability result in lower levels of residual variance, a critical component in estimation of genetic parameters.

### Phenotypic correlations with production traits

The positive phenotypic correlations between CH_4_, CO_2_, LW, and CW weight suggest that animals with heavier LW produce more CH_4_ and that reducing CH_4_ could lead to a negative effect on animal growth as the relationship between CH_4_ and LW was linear (Bird-Gardiner et al., 2017). While Bird-Gardiner et al. (2017) observed a stronger correlation (0.54–0.59 vs. 0.31 in the present study) between CH_4_ and LW, the LW definition varied between both studies from a mid-test period weight in Bird-Gardiner et al. (2017) to an end of test LW, prior to slaughter. Increased DMI intake is associated with higher levels of CH_4_ and CO_2_, and reduction of either trait will reduce DMI. Bird-Gardiner et al. (2017) also reported that the relationship between CH_4_ and DMI was both positive and linear, however, values reported were higher than this study (0.62–0.75 vs. 0.31). Methane was moderately correlated with DMI (0.31) in this study; hence, any CH_4_ mitigation strategy that directly targets reductions in CH_4_, without cognizance of DMI, may result in a reduction in DMI, which, in turn, may reduce animal productivity. Additionally, DMI had a strong positive correlation with LW (0.58) and a moderate correlation with CW (0.46), a trait of economic importance in current breeding goals. While no zero correlations were observed between CH_4_ and traits of economic importance, the correlations observed between RCH_4_ and other traits imply that reducing RCH_4_ as a mitigation strategy will not have an impact on DMI and, hence, animal productivity.

Phenotypic correlations between GEM system recorded traits (CH_4_ and CO_2_) are slightly weaker than correlations quoted in the literature to date, likely due to the size of the dataset, differences between sexes observed, and the multi-breed nature of this data set. Renand et al. (2019) observed a correlation of 0.86 and 0.83 between CH_4_ and CO_2_ in the beef heifer data studied, compared with a correlation of 0.60 in this study across sexes. Correlations observed in Smith et al. (2021) between CH_4_ and CO_2_ of 0.63 are similar to the 0.61 correlation observed in this study.

The inclusion of additional methane traits in the analysis was due to a lack of consensus in the literature for the goal trait in relation to CH_4_. Alternative definitions of CH_4_ are desirable for a number of reasons, including the potential of being a better metric for CH_4_, the potential use of proxy traits in the absence of CH_4_ measures and inclusion of CH_4_ in breeding goals. While some traits are ratio traits between economically important traits, alternative methane traits, independent of animal performance, need to be explored. Intensity traits (MIL and MIC) have strong correlations with each other (0.97), however, by definition are not the same, but may highlight the opportunity to include either LW or CW in a RCH_4_ trait. The strong correlations observed between the methane ratio traits (MY, MIL, MIC) are largely in concordance with existing literature (Herd et al., 2014; Renand et al., 2019; Richardson et al., 2021). The desire for utilization of ratio traits comes from the ease of calculation, ease of interpretation, and the ability to compare statistics across populations. However, these existing studies (Herd et al., 2014; Renand et al., 2019; Richardson et al., 2021) have highlighted the undesirable nature of using a ratio trait in future analysis, primarily due to the statistical properties of the trait leading to an unequal response to selection, and potential unfavorable correlations with traits of economic importance. Increased error variance as a proportion of total variance, strong correlations between ratio traits and the component traits, and the difficulty of determining the response to selection are all attributes of ratio traits that make usage undesirable (Berry et al., 2015). Residual CH_4_ had non-significant relationships with DMI and LW, which was not surprising, as the trait definition adjusted for both of these measures. Additionally, RCH_4_ had non-significant relationships with muscle scanning traits and carcass traits, which was explained by the relationship of these traits to DMI and LW. Residual CH_4_ presents some difficulty in relation to interpretability (Manzanilla-Pech et al., 2022), similar to ratio traits. End user interpretability of a CH_4_ trait is paramount to utilization and has presented some obstacles with residual feed intake (Manzanilla-Pech et al., 2022). The use of phenotypically derived RCH_4_ phenotypes in genetic evaluations may be problematic at a practical level where one or other of the underlying traits is unavailable. The construction of phenotypic RCH_4_ as defined in this paper requires availability of the gross CH_4_, DMI, and LW phenotypes, which may not be available in all populations where CH_4_ is recorded. In such scenarios, an alternative approach from a genetic evaluation perspective might be to evaluate the available gross traits and subsequently derive a genetic RCH_4_ using the resulting estimated breeding values.

While correlations between CH_4_ and both EMA and CC in this study were non-significant, the relationship between muscle traits and CH_4_ needs further analysis as previous work by Herd et al. (2014) estimated a phenotypic correlation between CH_4_ and EMA to be 0.29 ± 0.04, albeit using respiration chambers. FD correlations were similar between this study and Herd et al. (2014); however, no existing GEM system study has analyzed these ultrasound scanning traits in conjunction with CH_4_. The presence of phenotypic correlations between CH_4_ and performance traits suggests the need for further investigation in relation to potential genetic correlations. As mitigation strategies based on a reduction of RCH_4_ would not compromise animal productivity, RCH_4_ is a trait that provides the best opportunities for CH_4_ mitigation going forward.

## Conclusion

GEM systems successfully capture CH_4_ measurements at various hours throughout the day, however, they rely on frequent visitation by the animal. The repeatability of averaging periods suggests a possibility to use a protocol consisting of average CH_4_ and CO_2_ measurements based on a 10-d test period, however, it may be necessary to include all individual GEM system measurements in future genetic analysis to account for infrequent visitor animals to the GEM system. The data edits and 10-d averaging period evaluated in this study are now the proposed protocol used for GEM system measurement data handling for future genetic analysis of this data. The phenotypic correlations observed in this study demonstrate that animals with larger LW produce more CH_4_ and consume more dry matter feed in a commercial feedlot environment and while numerous methane traits exist and can be computed, RCH_4_ is independent of animal performance and provides the best opportunity with regard to mitigation strategies whereby CH_4_ is included in genetic evaluations.

## Supplementary Data

Supplementary data are available at *Journal of Animal Science* online.

Figure S1. Pen layout where animals undertook methane, carbon dioxide, and dry matter intake data recording. Three separate sheds were used in this study, with two replicate sheds based on layout A, each containing 4 pens and one shed with layout B, containing 4 pens; resulting in 12 pens in total.

skac349_suppl_Supplementary_Materials

## Abbreviations:

CC: carcass conformation
CF: carcass fat
CH_4_: methane
CO_2_: carbon dioxide
CW: carcass weight
DMI: dry matter intake
EMA: eye muscle area
FD: fat depth
GEM: GreenFeed emission monitoring
ICBF: Irish Cattle Breeding Federation
IMF: intramuscular fat
LW: live weight
MIC: methane intensity (carcass weight)
MIL: methane intensity (live weight)
MY: methane yield
RCH_4_: residual methane adjusted for dry matter intake and live weight
RFID: radio frequency identification
TMR: total mixed ration
