# Supplementary material for: Phenotypic relationship and repeatability of methane emissions and performance traits in beef cattle using a GreenFeed system
Source: J Anim Sci. 2022 Oct 21;100(12):skac349. doi: 10.1093/jas/skac349 (PMC9733524; doi:10.1093/jas/skac349)
Supplement: skac349_suppl_Supplementary_Materials [file skac349_suppl_supplementary_materials.docx]

**Supplementary materials**

Table S1. Breed description by sire and dam of animals included in the study.

| Animal Type | Beef x Beef | | Dairy x Beef | | Dairy | |
| --- | --- | --- | --- | --- | --- | --- |
| Number of intakes | 21 | | 9 | | 5 | |
| Number of animals | 730 | | 209 | | 142 | |
| Average Breed % | Sire breed | Dam breed | Sire breed | Dam breed | Sire breed | Dam breed |
| Angus | 13% | 12% | 46% |  |  |  |
| Aubrac | 4% | 1% | 6% |  |  |  |
| Belgian blue | 3% | 4% | 1% |  |  |  |
| Charolais | 22% | 13% |  |  |  |  |
| Holstein/ Freisian |  |  |  | 89% | 80% | 93% |
| Hereford | 2% | 6% | 30% |  |  |  |
| Jersey |  |  |  | 3% | 20% | 6% |
| Limousin | 27% | 37% | 14% |  |  |  |
| Piedmontese | 2% | 2% |  |  |  |  |
| Parthenaise | 2% | 1% |  |  |  |  |
| Saler | 7% | 3% |  |  |  |  |
| Shorthorn | 3% | 2% | 2% |  |  |  |
| Simmental | 12% | 18% |  |  |  |  |
| Other | 2% | 1% | 1% | 8% |  |  |


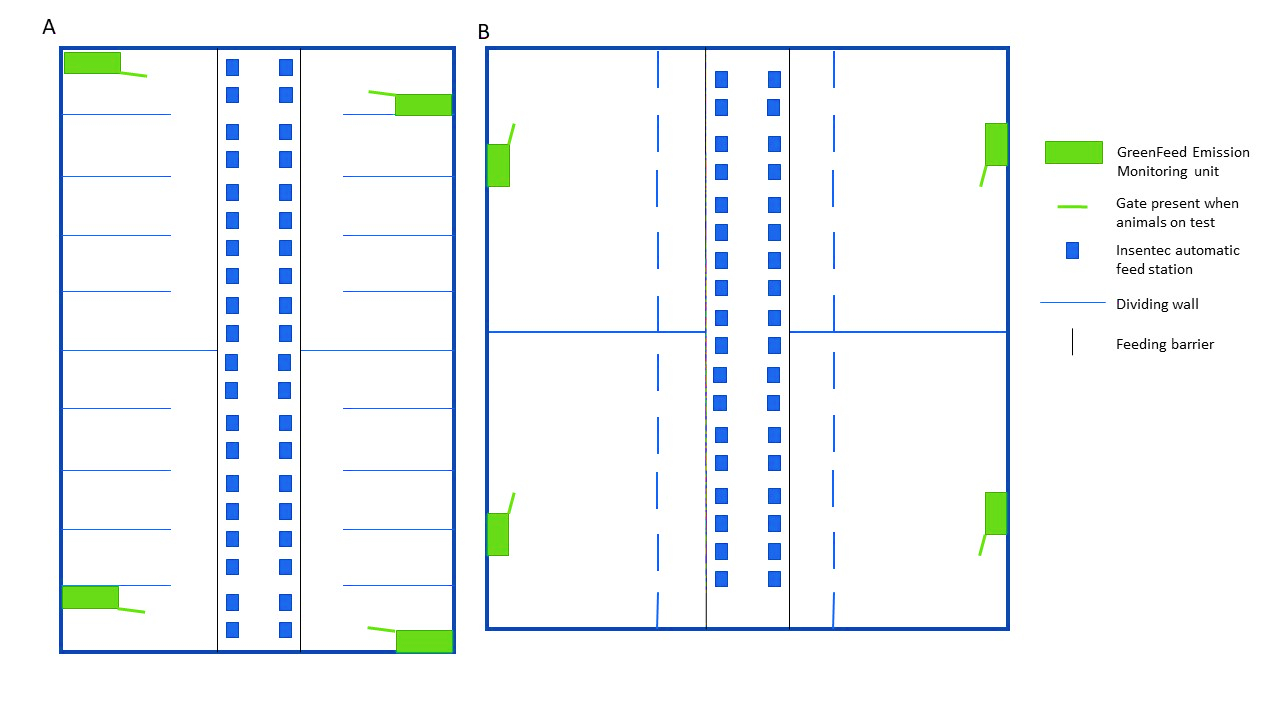


Figure S1. Pen layout where animals undertook methane, carbon dioxide and dry matter intake data recording. Three separate sheds were used in this study, with two replicate sheds based on layout A, each containing four pens and one shed with layout B, containing four pens; resulting in 12 pens in total.
